# Long-Term Activity-Dependent Plasticity of Action Potential Propagation Delay and Amplitude in Cortical Networks

**DOI:** 10.1371/journal.pone.0002088

**Published:** 2008-05-07

**Authors:** Douglas J. Bakkum, Zenas C. Chao, Steve M. Potter

**Affiliations:** Laboratory for Neuroengineering, Coulter Department of Biomedical Engineering, Georgia Institute of Technology and Emory University School of Medicine, Atlanta, Georgia, United States of America; Tel Aviv University, Israel

## Abstract

**Background:**

The precise temporal control of neuronal action potentials is essential for regulating many brain functions. From the viewpoint of a neuron, the specific timings of afferent input from the action potentials of its synaptic partners determines whether or not and when that neuron will fire its own action potential. Tuning such input would provide a powerful mechanism to adjust neuron function and in turn, that of the brain. However, axonal plasticity of action potential timing is counter to conventional notions of stable propagation and to the dominant theories of activity-dependent plasticity focusing on synaptic efficacies.

**Methodology/Principal Findings:**

Here we show the occurrence of activity-dependent plasticity of action potential propagation delays (up to 4 ms or 40% after minutes and 13 ms or 74% after hours) and amplitudes (up to 87%). We used a multi-electrode array to induce, detect, and track changes in propagation in multiple neurons while they adapted to different patterned stimuli in controlled neocortical networks in vitro. The changes did not occur when the same stimulation was repeated while blocking ionotropic gabaergic and glutamatergic receptors. Even though induction of changes in action potential timing and amplitude depended on synaptic transmission, the expression of these changes persisted in the presence of the synaptic receptor blockers.

**Conclusions/Significance:**

We conclude that, along with changes in synaptic efficacy, propagation plasticity provides a cellular mechanism to tune neuronal network function in vitro and potentially learning and memory in the brain.

## Introduction

The specific arrival times of afferent synaptic excitatory and inhibitory potentials determine how they summate as they converge towards a neuron's soma and whether or not and when to fire an action potential. Tuning the timing of such input would provide a powerful mechanism to adjust the output of a neuron, and potentially, could be a mechanism for learning and memory in the brain. We hypothesized that axonal plasticity of action potential propagation could vary how information is processed in the brain by regulating the timing and amplitude of synaptic input impinging on a neuron. This is fundamentally different than the dominant theories of neural plasticity that focus on the efficacy of synaptic transmission.

Axons in the mammalian cortex have traditionally been regarded as stable transmission cables. However, this view is more likely due to a lack of, rather than support from, experimental evidence [1], [2] because their small diameter (<1 µm) makes direct recordings at multiple sites difficult. The action potential is often viewed as a binary signal, but recent experiments have found a novel analog mechanism: the ability to encode a neuron's background synaptic activity in the amplitude of its action potential in cortical slices from ferrets [3] and hippocampal slices from rats [4]. Moreover, the temporal control of action potential propagation could encode a vast amount of information. For example, introducing non-uniform, although fixed, conduction delays in a model network produced a potentially unlimited number of “polychronous” groups of neurons capable of recognizing and classifying complex spatiotemporal stimuli [5], a theoretical canvas for memories; spike-timing dependent plasticity (STDP) organized the groups by potentiating afferent signals whose timings were close enough to integrate and produce an action potential in a post-synaptic neuron. Alternatively, modulating the propagation speeds themselves could also compose such groups [6]. STDP causes long-term synaptic potentiation or depression based on the temporal order of pre and post-synaptic activation [7], and the magnitude of plasticity is greatest at smaller activation intervals, with a sharp discontinuity from potentiation to depression as the post-synaptic neuron went from lagging to leading. This feature of the STDP rule makes it a sensitive detector of relative spike order and a means to create and maintain causal pathways or synfire chains [8]. Finely tuning action potential propagation speed could provide a means to continuously mold functional circuits in the brain throughout life.

The precise control of action potential propagation has been shown to be important in both the central and peripheral nervous systems after development or many days of experience [9], [10], but modulation of propagation has not been reported on the seconds to hours time scales relevant to learning and memory consolidation. For instance, the propagation of action potentials in the olivocerebellar pathway of rats can vary by 40% such that isochronic conduction occurred between neurons independent of axon length, a finding conserved even between animals [11]. In addition, a decrease in the size of a spinal stretch reflex (H-reflex) after 40 days of operant conditioning was accompanied by an 8% (rat) or 6% (monkey) decrease in motoneuron conduction velocity [12]. The reported propagation velocities were consistent with myelinated fibers. Propagation speeds have been proposed to be tuned with millisecond precision through axon diameter [11], [13], myelin thickness [13], [14], the location of nodes of Ranvier [15], and the kinetics of voltage gated sodium channels [16].

Research on neural intrinsic excitability has demonstrated that, in addition to the well studied synaptic plasticity, activity-dependent plasticity can be expressed outside of synapses on fast time scales [1], [17]–[19]. However, the proposed molecular mechanisms involved have not been investigated with respect to plasticity of action potential propagation. In embryonic rat hippocampal cultures, injecting depolarizing currents into a post-synaptic neuron such that it fired in synchrony with pre-synaptic activity for 80 seconds caused long-term plasticity of the excitability of the pre-synaptic neuron, requiring post-synaptic NMDA-R and pre-synaptic PKC activation and affecting the gating kinetics of voltage-gated sodium channels [20]; while in part synaptic in origin, the plasticity was expressed outside of synapses. These channels actively propagate action potentials, and changing their gating kinetics could change action potential shape and propagation velocity within the central nervous system within minutes.

The above work demonstrates that action potential propagation can encode information [5], [21], is regulated [9]–[11], and previously described mechanisms [20] could result in its plasticity. To our knowledge, however, these mechanisms have not been related to propagation plasticity, and propagation has not been shown to be regulated on the minutes to hours time scale relevant to much learning and memory. Here, we show experimental evidence of the rapid induction of activity-dependent long-term plasticity of action potential propagation. Using networks of rat neocortical neurons and glia grown over multi-electrode arrays (MEA), we observed the timing of direct electrically-evoked action potentials (dAPs) in multiple neurons changed up to 4 milliseconds (40%) after minutes of recording and up to 13 milliseconds (74%) after hours, adapting to different patterns of low frequency stimulation. The estimated propagation velocities suggest propagation occurred in unmyelinated axons [1], [9], [10], [22], [23]. The plasticity was activity-dependent since no change occurred when synaptically-evoked action potentials (sAPs) were blocked using antagonists of the fast synaptic transmitter receptors NMDA-R, AMPA-R, and GABA-R. As with the intrinsic excitability experiments, the plasticity was expressed outside of the synapses. Action potential amplitude similarly adapted to the various patterned stimuli (up to 87% change). We conclude that propagation plasticity is a cellular mechanism used to tune temporal coding schemes and information processing in neural networks, and potentially learning and memory in the brain.

## Results

**Figure 1:**
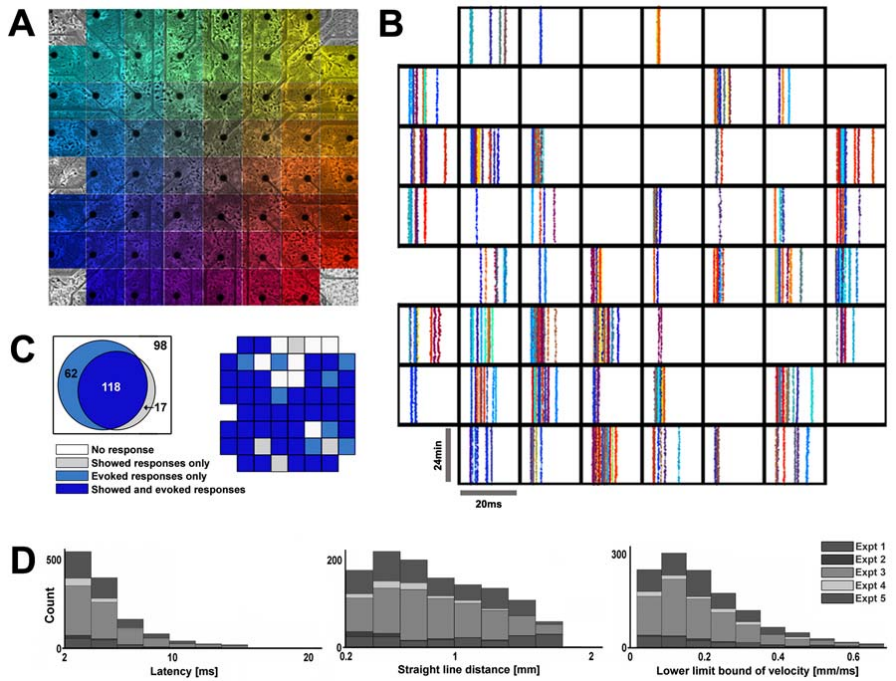
Multi-electrode arrays (MEA) robustly detected dAPs. (A) Neurons at 6 days in vitro grown over an MEA with 30 µm diameter electrodes spaced 0.2 mm apart. The large reference electrode is outside the field of view. Color represents the location of the stimulation electrode that evoked the dAPs plotted in B. (B) Recording electrodes (arranged topographically) detected dAPs (dots) in a 4 week-old culture in abundance. (C) Venn diagram comparing the proportion of the electrodes showing and/or evoking dAPs in 5 three to four week-old cultures from 3 dissociations (295 electrodes; data also in Fig. 5). Their incomplete overlap suggests that the location of an extracellularly recorded action potential can differ from the location of an extracellularly evoked action potential within a given neuron. The data from B are presented in the adjacent plot as an example. (D) Histograms of dAP latencies, distances from the stimuli site, and estimated velocities.

**Figure 2:**
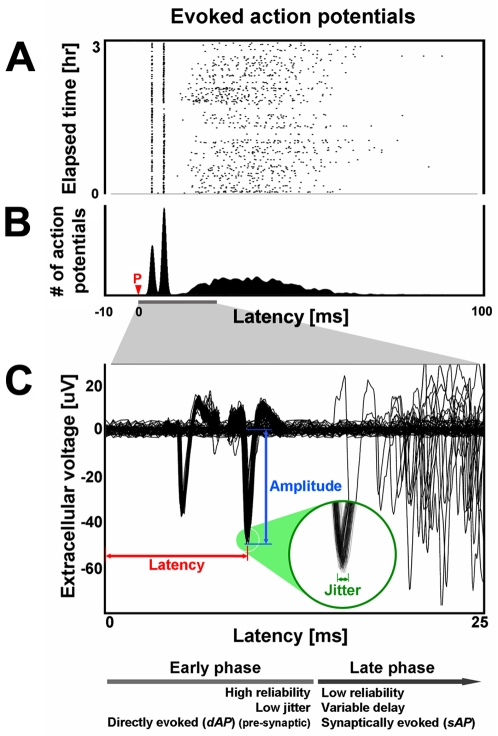
Electrically evoked neural activity. Action potentials recorded on one extracellular electrode in response to stimulation at another consist of an early directly-evoked action potential (dAP) phase and a later synaptically-evoked action potential (sAP) phase. (A) The raster plot (1 dot per action potential) shows the first 100 ms of neural responses to 3 hours of periodic 1/2 Hz probe stimulation (red P). (B) The peri-stimulus time histogram and (C) overlaid extracellular voltage traces across all trials emphasize the consistency of the early phase with respect to the later phase. The sharp peaks in the histogram arise from two dAPs. See also [26].

**Figure 3:**
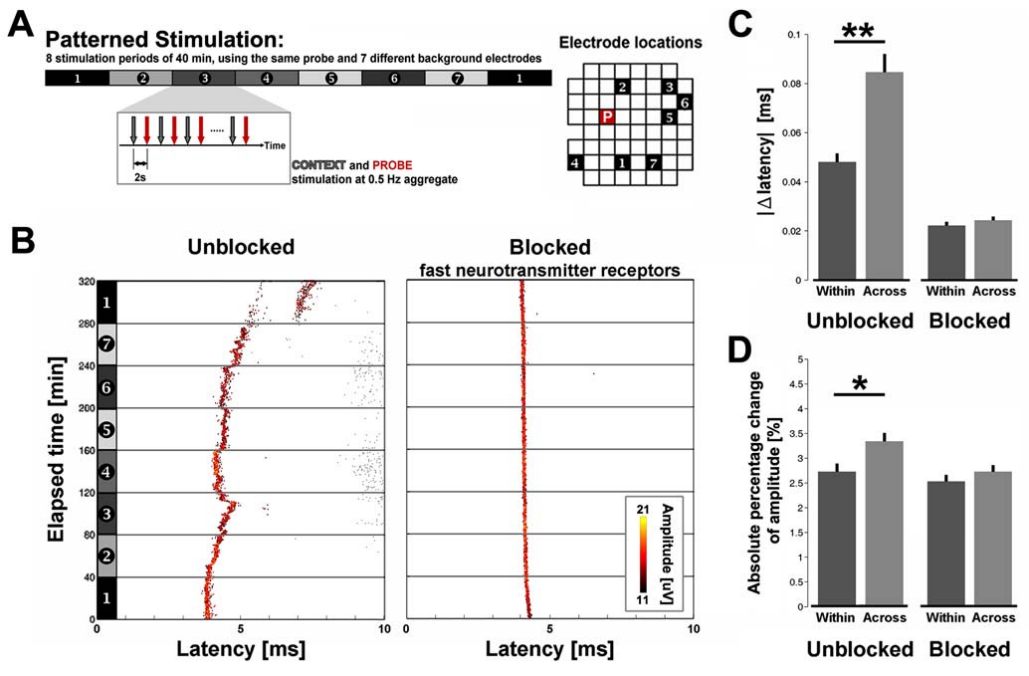
Action potential propagation depended on ongoing neural activity and stimulation pattern. (A) Experiment protocol. 1/4 Hz probe stimuli (red arrows) produced dAPs whose latencies and amplitudes were investigated for plasticity. A context electrode (gray arrows) was stimulated 2 sec prior to each probe stimulus, giving an overall 1/2 Hz stimulation frequency, and its location was shifted every 40 min to produce different patterns of stimulation (numbers and shaded bars). Right: electrode locations for data in B. (B) Example raster plots of a given dAP recorded on one electrode in response to probe stimulation of another electrode in culture media (left, Unblocked; sAPs are plotted with smaller markers) and when blocking sAPs (right, Blocked). Ongoing neural activity modified latency (*x* axis) and amplitude (color). Varying stimulation pattern (Across) significantly altered (C) dAP latency (**P<1e-6, Wilcoxon signed rank test for paired samples. Unblocked: *n* = 130 dAP trains; Blocked: *n* = 115 dAP trains. 6 cultures from 4 dissociations) and (D) amplitude (*P = 0.003) within 5 minutes (mean+s.e.m.). See Results.

**Figure 4:**
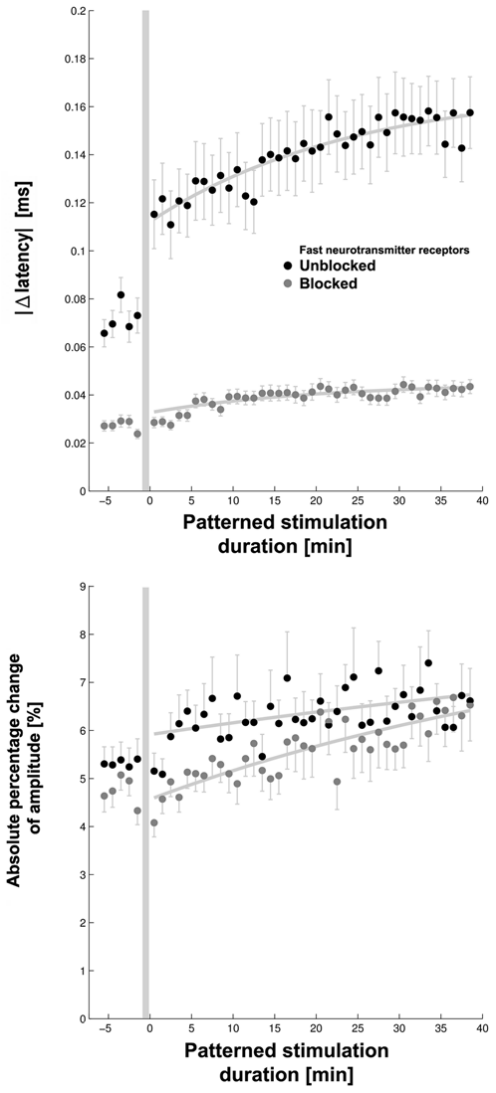
Changes in dAP propagation accumulated throughout the 40 minute duration of a patterned stimulation. DAP latencies (Top) and amplitudes (Bottom), from all experiments in Figure 3, were averaged over 1 minute time-bins and compared to those during the 1 minute time-bin just prior to shifting the location of the context electrode (shaded area). The absolute values of the changes in propagation were then averaged (dots; mean±s.e.m.). Exponential curves (thick lines) fit to the data indicate the rates of adaptation. Blocking sAPs minimized the effect of shifting the patterned stimulation that occurred at time = 0 min).

**Figure 5:**
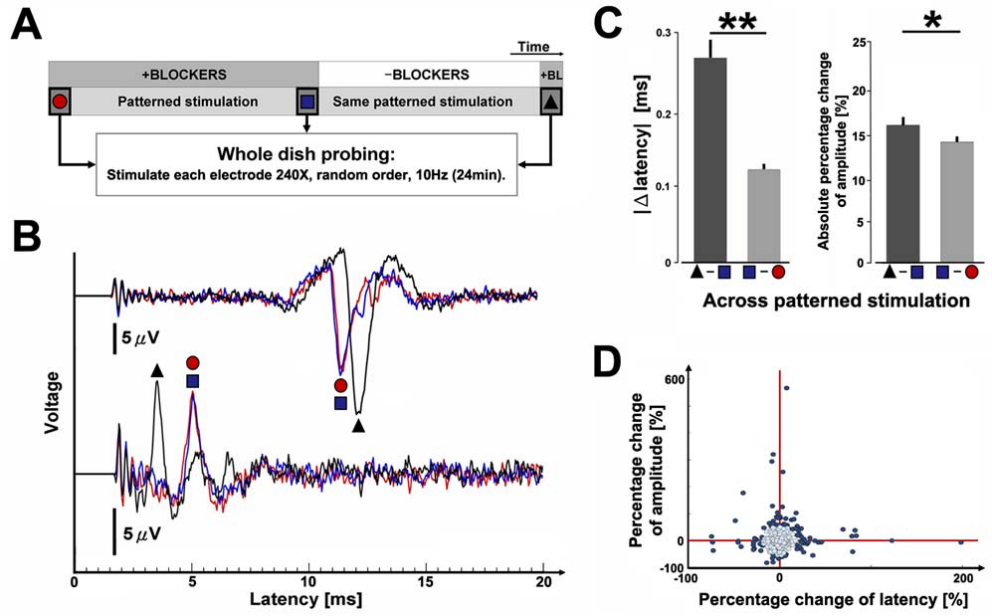
The cause of plasticity was neural activity, and the site of plasticity was not synaptic. (A) Experiment protocol. SAPs were blocked to eliminate the influence of ongoing synaptic activity, and 3 identical periods of whole-dish probing (shape and color coded) were applied before and after the 5 hours and 20 minutes of patterned stimulation (Fig. 3A). (B) Example extracellular voltage traces for two separate dAPs during each whole-dish probing period (240 traces averaged). Changes that accrued during the patterned stimulation persisted (blue square to black triangle): they were not reflections of ongoing synaptic activity. Changes were minimal during patterned stimulation in the presence of blockers (red circle to blue square): the accrued changes were not artifacts from the electrical stimulation or replacing media. (C) Statistics for all observations (mean+s.e.m. **P<1e-6 and *P = 0.003. Wilcoxon signed rank test for paired samples. *n* = 904 dAP trains. 5 cultures from 3 dissociations). (D) Changes in latency were not monotonically correlated to changes in amplitude (*P* = 0.22, *ρ* = 0.04; Spearman's rank correlation coefficient). The outlying data points, using an arbitrary cut-off at 10% of the distribution, were plotted with darker dots.

This section begins with data describing the ability of our preparation to characterize dAP propagation (Figs. 1 and 2). This is followed by experimental evidence of changes in the latencies and amplitudes of the dAPs in response to patterned stimulation (Figs. 3, 4, and 5). Control experiments indicated that the plasticity, while requiring sAPs to be induced, was expressed independently of synaptic activity since changes persisted when sAPs were blocked. The section ends with a theoretical consideration of our results intended to inspire others to explore the potential roles of action potential propagation in neural computation.

### Propagation of direct electrically-evoked action potentials (dAPs) was robustly detected using planar multi-electrode arrays (MEAs)

By using cortical neurons cultured on extracellular multi-electrode arrays (MEAs) (Fig. 1A and Methods), action potentials can be sampled at multiple sites up to millimeters apart for extended durations. Stimulation by one electrode evokes neural responses that can be recorded in a subset of the rest of the electrodes (Figs. 1B and 2). Of these, dAPs have been observed up to 25 ms later and can be distinguished from subsequent sAPs based on their high reliability of occurrence (>80%), low jitter (160 µs), and consistency of waveform across trials [24]–[26] (Fig 2; see Methods for a description of automated dAP detection). They are pre-synaptic as they persist when synaptic activity is blocked using fast neurotransmitter receptor antagonists of NMDA-R, AMPA-R, and GABA-R, and they are propagating action potentials as they are eliminated with TTX [26]. In the following experiments, we quantified changes in dAP propagation by measuring their amplitudes and latencies (Fig. 2C) after the downswing of a biphasic stimulus pulse, termed a “probe”.

Figure 1 shows that MEAs can be used to investigate the dAPs of many neurons in a cultured network. Our MEAs contained 59 functional electrodes (Fig. 1A), and many could evoke and/or record neural activity depending on the relative location of the neurons (Fig. 1B). On average, an electrode that evoked activity yielded 6.2±5.7 dAPs (mean±std) detected elsewhere. Figure 1B contains a set of raster plots of dAP latencies (*x axes*), arranged topographically by recording electrode and color coded by stimulation electrode, in response to 24 minutes (*y axes*) of “whole-dish probing” of a 4 week old culture. Whole-dish probing consisted of stimulating each electrode 240 times in random order at an overall rate of 10 Hz, with sAPs blocked. It was used here to quickly sample all accessible dAPs in each network and later to quantify induced plasticity.

Extracellular stimulation and recording of neural activity may act on different sites of a neuron [27]. Interestingly, in data from 5 cultures (Figure 1C), 34% of the stimulating electrodes that could evoke activity (180 electrodes) did not record activity (62 electrodes), and 13% of the electrodes that recorded activity (135 electrodes) did not evoke activity (17 electrodes) when stimulated. Although specific numbers will depend on the density and location of the neurons (see Methods), this nevertheless suggests that action potentials tended to be recorded from different parts of a neuron than where they were induced by electrical stimulation. For example, an action potential in a soma may produce a larger signal on an extracellular electrode than one in an axon because more ions would be needed to depolarize the larger surface area. Therefore, the recording range of an electrode would be larger for cell bodies than axons. Conversely, an axon would require less current than a soma to become depolarized, and the stimulation range of an electrode would be larger for axons than cell bodies.

Figure 1D further characterizes the observed dAPs. A few were detected up to 25 ms after being evoked, but the majority were earlier. They could not be detected sooner than about 2 ms nor on the stimulating electrode due to the presence of electrical stimulation artifact [28]. DAPs could have been evoked in the middle of axons that passed near an electrode, and thus the actual delay from the neuron to its post-synaptic targets would be longer than what was measured. Moreover, increased electrode spacing may lead to longer detectable latencies. Due to the geometry of the MEA, the majority of distances between stimulating and recording electrodes were closer to the minimum distance of 0.2 mm. Therefore, the plotted histogram of distances was normalized by the distribution of all possible inter-electrode distances. The estimated velocities suggested the detected dAPs propagated through unmyelinated neurites [1], [9], [10], [22], [23].

### Action potential propagation depended on neural activity and variation of stimulation pattern

By varying a simple low frequency stimulation pattern every 40 minutes, we induced changes in the time elapsed for dAPs to propagate from a probe electrode to a recording electrode and also in their extracellularly recorded amplitude (Fig. 3). Each pattern consisted of alternatively stimulating 2 electrodes at 2 second intervals. (1/4 Hz stimulation per electrode and 1/2 Hz overall stimulation; Fig. 3A). The second electrode, termed probe, was fixed at one site and used throughout the duration of an experiment to consistently sample dAPs. The location of the first “context” electrode was moved spatially every 40 minutes to make each new pattern. The dAPs evoked by context stimuli were not analyzed. Interestingly, we found that the dAPs evoked by the probe stimuli changed via gradual shifts and jumps in latency (up to 4 ms or 40%) and amplitude (up to 20 µV or 80%), but not when the stimulation was repeated in the presence of antagonists of NMDA-R, AMPA-R, and GABA-R (Fig. 3B). These blocked all spontaneous activity (sAPs) except for an occasional self-firing neuron [29]. Therefore, even though dAPs were detected pre-synaptically, changes in their latency and amplitude required successful synaptic transmission of glutamate and/or GABA. Electrical artifact and chemical interactions at the electrode interface did not contribute since changes were minimal for identical stimulation while blocking sAPs (Fig. 3). Jumps in latency (Fig. 3B after 280 min) may reflect the occurrence of microsaltatory conduction [30].

The propagation of dAPs adapted to new stimulation patterns within minutes. The absolute changes in dAP latencies were significantly greater just after a change in the patterned stimulation than at the end of the 40 minute interval of a patterned stimulation (Fig. 3C; **P<1e-6). When sAPs were blocked, a variation in stimulation pattern had no effect (*P* = 0.24). To a lesser significance, dAP amplitudes were similarly altered (Fig. 3D; *P = 0.003), but not when sAPs were blocked (*P* = 0.59). The change “across” adjacent patterned stimulation intervals was calculated between the 5 minute periods just prior to and just after shifting the location of the context electrode. Each 5 minute period consisted of 75 probe stimuli. The change “within” a period was calculated between the 10-to-5 minute and the 5-to-0 minute periods prior to a shift. Detected action potential amplitudes, and subsequently the magnitude of changes in amplitude, are proportional to the distance from a recording electrode. Thus amplitude measurements from different dAPs were made comparable by using absolute *percentage* change.

All dAPs did not necessarily undergo changes in propagation, which tempered the averages in Fig. 3C & D. The statistics above were calculated using all dAPs in order to provide robust support for our conclusions, but analyzing the upper tail of the distribution in changes can give perspective on the magnitude of the plasticity. For example, when considering the 10% of the dAPs with the largest change in latency, the mean of the absolute changes in latency across and within patterned stimulation intervals was 0.42 ms per 5 min and 0.09 ms per 5 min, respectively. For the 10% of the dAPs with the largest percentage change in amplitude, the mean of the absolute percentage changes was 11% and 4% for across and within patterned stimulation intervals, respectively. The patterned stimulation protocol could be considered a simplified analog for memory processes found in the brain. For example, repetitive cortical activation by the hippocampus during sleep consolidates memories [31] and repetitive body movements lead to cortical plasticity [32].

Changes in dAP latencies and amplitudes were largest after shifting the patterned stimulation and continued to accumulate over time (Fig. 4). With sAPs blocked, the variability in dAP propagation decreased and shifting of the patterned stimulation appeared no longer to have an effect. This further demonstrated that propagation plasticity depended on the presence of neural network activity (sAPs) and was enhanced by varying patterned stimulation. Adaptation of latencies to a 40 minute interval of patterned stimulation, without blocking sAPs, was shown by a decrease in the rate of change, which was fit by an exponential curve with a time constant *τ* of 24.3 minutes (*P*<1e-6, *R^2^* = 0.78). Change in amplitudes had a time constant of 104.2 minutes (*P* = 0.002, *R^2^* = 0.23). The decay in plasticity suggests that action potential propagation may have been habituating to individual patterned stimuli. With blockers, the time courses of changes in latencies and amplitudes were not constant, which indicated the presence of a baseline plasticity. This may have arisen from an individual neuron's response to the electrical stimuli or in turn from homeostatic mechanisms regulating neural ion channels in response to suppressed neural activity [33], [34].

### Plasticity of action potential propagation had a non-synaptic expression

Although induction of propagation plasticity depended on the occurrence of sAPs, long-term plasticity of action potential latency and amplitude was expressed outside of synapses (Fig. 5). (Accompanying plasticity expressed at synapses is expected to have occurred also.) The changes in dAP latencies and amplitudes in Figures 3 and 4 could reflect a variation in propagation or instead a transient response to a variation in the recent background synaptic activity. The technique of using extracellular electrodes to investigate axonal properties has a long history, and delays in antidromic propagation have been shown to depend on the somatic membrane potential [24]. In particular, the impedance mismatch due to the change in volume from the axon into the soma causes a delay in propagation proportional to the somatic membrane potential, which varies with synaptic input [24]. However, such changes in delay were elastic, recovering within 100 ms after altering the membrane potential.

To experimentally eliminate variation in membrane potential due to recent synaptic activity, additional whole-dish probing periods were conducted, in the presence of fast synaptic receptor blockers, before and after the patterned stimulation protocols (Fig. 5A), and long-term plasticity of action potential propagation delay and amplitude persisted (Fig. 5B). The whole-dish probing periods consisted of stimulating every electrode 240 times in random order at an overall frequency of 10 Hz (24 minutes) and recording the dAPs. If the changes in propagation induced by the patterned stimulation were expressed independently of synaptic activity (and were not transient), dAP latencies and amplitudes would vary between the periods of whole-dish probing before and after the 5 hour and 20 minute sequence of patterned stimulation intervals. Then as a control, dAP latencies and amplitudes should not vary between the periods of whole-dish probing before and after patterned stimulation conducted with blockers, where plasticity did not occur. Consequently, action potential propagation did change (in one case, latency decreased by 13 ms or 74%) significantly more after the patterned stimulation without blockers than with blockers (Fig. 5C; **P<1e-6 for change in latency, *P = 0.003 for change in amplitude). Propagation plasticity was expressed outside of the synapses since it could be detected in the presence of synaptic blockers. Not all dAPs changed latency and amplitude (Fig. 5D), suggesting the plasticity induced by the patterned stimuli discriminated among different pathways of propagating neural activity [35]. When considering the 10% of the dAPs with the greatest change in latency, the median of the absolute changes in latency across patterned stimulation without blockers (Fig. 5C) was 0.80 ms. The change was 0.12 ms for the corresponding dAPs across patterned stimulation with blockers. For the 10% of the dAPs with the largest percentage change in amplitude, the median of the absolute percentage changes in amplitude across patterned stimulation without blockers was 48%. The change was 19% across the patterned stimulation with blockers. As opposed to Figs. 3C and 3D which showed changes across 5 minutes, the propagation plasticity averaged in Fig. 5C and plotted in Fig. 5D had accrued over the duration of the entire patterned stimulation protocol, 5 hours and 20 minutes.

Increasing the overall stimulation frequency from 1/2 Hz, as with patterned stimulation, to 10 Hz during the whole-dish probing was done to decrease experiment durations. Using a different stimulation frequency was not of concern because the whole-dish probing was always done in the presence of blockers, where propagation plasticity was minimal, and dAPs evoked by whole-dish probing sequences were compared only to those evoked by other whole-dish probing sequences. Moreover, the per electrode stimulation frequencies were comparable: whole-dish probing stimulated a given electrode every 5.9 seconds on average versus every 4 seconds during patterned stimulation.

### Theoretical computational capacity

A given neuron might have the ability to differentially modulate the timing and/or amplitude of action potentials impinging on multiple post-synaptic cells, greatly increasing the available computational capacity of a network. Both increases and decreases in dAP latency (up to 200%) and amplitude (up to 600%) occurred, but interestingly, a monotonic correlation between the two was not found (Fig. 5D; *P* = 0.22). Homogeneous plasticity of cell properties along the length of a neurite, for example of voltage gated Na+ channels, would be expected to cause a correlated change between action potential latency and amplitude. Therefore, the plasticity occurred either via different mechanisms or in a locally discriminate manner throughout the neuron, perhaps by geometrical variations in axonal varicosities which could cause conduction delays up to 100 s of µs at each synaptic bouton [22], [36].

As an example of the potential computational capacity for such a locally controllable propagation, a 1.2 mm axonal branch could achieve 1 billion temporal configurations for its synaptic outputs. Estimating the average conduction velocity to be 0.25 mm/ms (Fig. 1D, histogram peak multiplied by a safety factor of 2) and the discrete time resolution to be the average dAP jitter during patterned stimulation experiments without blockers, 160 µs, gives a discrete spatial resolution of 40 µm (*distance* = *velocity* * *time*). This spatial resolution would not be decreased by considering the magnitude of the observed latency changes nor by synapse density: axonal synaptic boutons were spaced 5 to 10 µm apart on average in cat cortical neurons [37]. Thus, a 1.2 mm length neurite would have 30 (1.2 mm divided by 40 µm) discrete loci of change, or over a billion (2^30^) possible temporal configurations available to its multiple synaptic targets in a neural network. A neuron would have even more capacity considering (1) temporal encoding is analog and not binary, (2) axonal arbors have 2 to 3 orders of magnitude greater length [1], [37] and (3) extensive branching [1], [38], (4) the capacity of separate branches are multiplicative, and (5) encoding via action potential amplitude was not considered. However, the actual temporal and spatial resolutions are not known, neither are the rules of plasticity induction, and computational capacity is only an upper limit to memory capacity. In particular, what governs an increase versus a decrease in latency and amplitude? The fact that changes in delay were bi-directional suggests that a rule does exist. An understanding of these rules is needed to apply propagation plasticity in computational models or in further exploring its role in cognition.

## Discussion

By accessing networks of neurons at multiple locations in both cultures and slice preparations, MEAs have already provided fundamental insights into neural information processing. For example, the propagation of neural activity in organotypic and acute rat cortical slices was found to obey a power law, optimizing the amount of information transmitted while preventing runaway network excitation [39]. Electrical stimulation of rat cortical cultures has induced plasticity specific to pathways [34] and regions [40] of neurons, suggesting plasticity rules exist at the network level. Recently, MEAs demonstrated that local chemical stimuli, in addition to electrical stimuli, could alter network activity states [41]. Furthermore, by utilizing MEA recordings to determine the feedback of subsequent stimulation patterns, closed-loop systems can be created to investigate “learning in vitro” [42]–[44]. The first report of learning used electrical stimuli to train a neural response to occur within a predetermined time interval [45]. Later, adaptive goal-directed behavior was observed in a simulated network [46] and applied to cultured neurons controlling a robotic drawing machine [47].

Here, we have identified plasticity mechanisms that depend on synaptic transmission for induction, but are nevertheless expressed without it. As with changes in synaptic strengths, the changes we observed in action potential propagation are also likely to influence computation, learning, and memory in neural systems. For example, changes in action potential delay alter the type and number of attractor states in recurrent delayed neural loops [48], [49] and neural networks [5]. Elucidating the cellular mechanisms of propagation plasticity is left to future work, but possibilities include non-uniform changes in ion channel properties [20], in the geometry of varicosities and branch points [36] or axonal arbors, in the proximity of glia [50], and in lipid membrane composition [51]. The changes we observed in cortical neurons could be generalized to occur throughout the brain, although the role of glial sheaths may dominate in faster-conducting myelinated axons [50]. Gap junctions were not considered because, in a similar preparation, both electrical and dye coupling experiments did not reveal coupled neurons [52]. Patterning axon growth over a series of electrodes [53] or nanowire transistor recording devices [23] and/or optical imaging [54] could expose the discrimination, resolution, and possible morphological correlates of propagation plasticity.

Past research has set the stage to discover the rules governing temporal coding in the brain. The neural orchestra is comprised of not only synapses but many instruments, in part tuned by propagation plasticity. By using an MEA to robustly detect and track changes in the propagation of electrically evoked action potentials, we found that variation of a low frequency patterned stimulation modulated action potential propagation delays and amplitudes. Even though the induction of plasticity depended on synaptically evoked action potentials, its expression was non-synaptic: action potential propagation. Propagation varied for different stimulation patterns and became more stable for unvarying patterns, attributes necessary for playing a role in encoding memories. Latencies and amplitudes increased and decreased in an un-correlated manner, potentially allowing a neuron to have variable synaptic transmission among multiple post-synaptic neurons. In summary, the results suggest that propagation plasticity could serve as a cellular mechanism to tune temporal coding schemes and information processing in neural networks. Plasticity mechanisms that regulate the timing and amplitude of synaptic input impinging on a neuron challenge the dogma that memories are stored solely as changes in synaptic efficacy.

## Materials and Methods

### Cell culture

We have developed techniques to maintain neural cultures and conduct experiments for many months [42]. Briefly, 50k cells from E18 rat cortices were dissociated using papain with trituration and plated over an approximately 3 mm diameter area on top of multi-electrode arrays (MEA; from Multi Channel Systems). A thin layer of polyethyleneimine followed by a 15 µL drop of laminin were used for cell adhesion. The cultures were grown in 1 mL of DMEM containing 10% horse serum with glutamax, insulin, and sodium pyruvate additives. Cultures matured for 3 to 4 weeks prior to experimentation. Experiments were conducted inside an incubator to control environmental conditions (35°C, 65% humidity, 9% O_2_, 5%CO_2_). The MEAs were sealed with a hydrophobic membrane (fluorinated ethylene–propylene) that is selectively permeable to O_2_ and CO_2_, and relatively impermeable to water vapor, bacteria, and fungus, allowing us to conduct long-term, non-invasive experiments. Extracellular MEAs were chosen over intracellular electrodes for multiple reasons. Experiments lasted up to 16 hrs; intracellular electrodes change a cell's physiology by perforation and by the introduction of the patch pipette solution, ultimately leading to cell death within at most a few hours. Additionally, recording the same neuron at multiple sites is difficult intracellularly but simple, robust, and non-destructive with MEAs (Fig. 1).

### Pharmacology

To block synaptically-evoked action potentials (sAP), fast synaptic receptor antagonists were applied at concentrations of 50 µM bicuculline methiodide (BMI), 100 µM 2-amino-5-phosphonovaleric acid (APV), and 10 µM 6-cyano-7-nitroquinoxaline-2, 3-dione (CNQX) (from Sigma), dissolved in culture medium and stored at −80°C. These are antagonists of GABA-R, NMDA-R, and AMPA-R, respectively. Fresh 35°C culture medium was used at the start of experiments and when changing medium between patterned stimulation and whole-dish probing experiments. When replacing medium from with to without the antagonists, cultures were washed 4 times by applying and discarding 1 mL volumes of fresh medium. A medium change lasted a couple minutes and cultures were allowed to equilibrate for an additional 30 minutes prior to beginning stimulation.

### Electrical stimulation and data acquisition

Electrically evoked activity was induced using symmetric positive then negative voltage pulses of 400 µs duration and 500 mV magnitude per phase [26] using a custom built all-channel stimulation circuit board [55] attached to the MEAs. Each MEA had 59 functional electrodes and 1 large ground electrode (reference) to the side (Fig. 1A). Data was collected through Multi Channel Systems' pre-amplifier and data acquisition card (MCCard), which had a 25 kHz sampling frequency and could accurately record microvolt signals. Data processing, visualization, artifact suppression, and spike detection were controlled using Meabench [43], [56]. Artifact suppression allowed us to detect dAPs 2 ms after being evoked [28]. Neural action potentials were detected if the absolute value of a voltage spike exceeded 5 standard deviations rms noise in amplitude.

**Figure 6:**
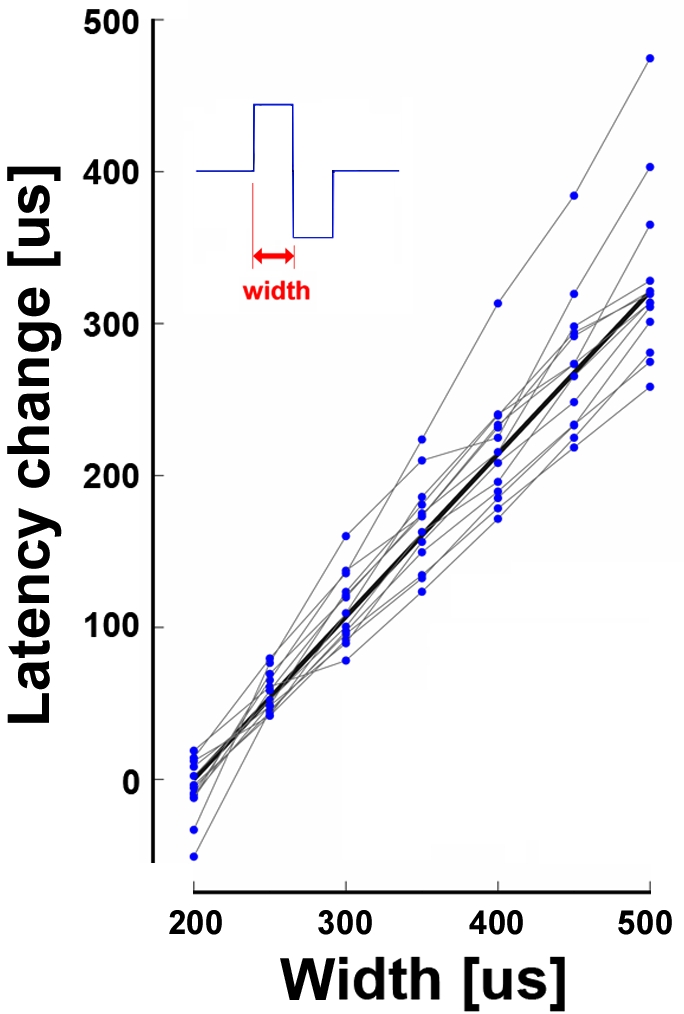
DAPs were time-locked to the downswing of biphasic voltage stimuli. Latencies of dAPs from 13 stimulation-electrode/recording-electrode pairs (thin lines) were measured from the beginning of voltage stimuli with various pulse widths (inset); sAPs were blocked. The thick line is a linear regression on all data points.

By recording extracellular voltage spikes of dAPs, an MEA is able to measure propagation delays with high precision (Fig. 6) and can reveal whether or not plasticity occurred after an experimental manipulation. Negative extracellular current most effectively depolarizes a neuron, which for a voltage step occurs during the pulse downswing (I = C*dV/dt). Thus, unlike during a current step, dAP timing can be precisely time-locked to the stimulus downswing, as confirmed by plotting dAP latency for different voltage step widths (Fig. 6) [26]. In Figure 6, the width of rectangular stimuli were varied while magnitude was kept constant at ±500 µV; 30 stimuli were delivered per width in random order. A linear regression of the averaged latencies (dots) from each of the 13 pairs was used to align the data: the regressions' y-intercepts at the 200 µV phase width were set as 0 µs latency change. Another linear regression on all aligned data points produced a representative slope of 1.10 µs/µs, indicating most dAPs were time-locked to the voltage downswing. A slope *m* = 0 would indicate the dAPs were triggered at the beginning of stimuli; *m* = 1, at the downswing; *m* = 2, at the end; and 0<*m*<1 or 1<*m*<2, in the middle of a phase. See also [26].

### Experiment parameters

For the patterned stimulation experiments, a 40 minute duration was chosen for each stimulation pattern to allow enough time to stabilize plasticity induced after the patterns were changed. A slow 1/2 Hz overall stimulation rate was chosen to avoid network fatigue or refractory periods [57] from compromising the evoked responses. The stimulation electrode evoking the most dAPs was chosen as the probe electrode. The probe was paired with electrodes evoking varying degrees of neural activity to create patterned stimulation with diverse network activity responses.

### Automated detection of direct electrically-evoked action potentials (dAPs)

**Figure 7:**
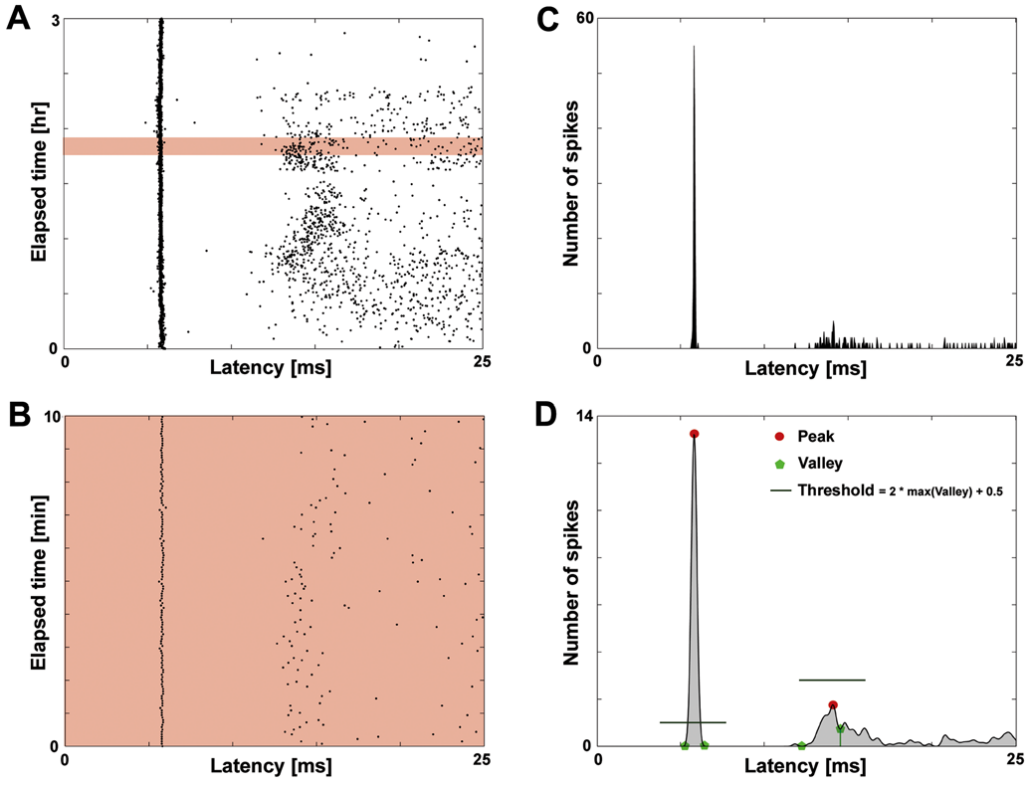
Detecting dAPs. DAPs were automatically detected and tracked from peaks and valleys in consecutive smoothed post-stimulus time histograms. (A) The first 25 ms of neural activity in response to 1/4 Hz probe stimulation were searched for the occurrence of dAPs in 10 min windows (shaded). (B) Expanded view of the shaded 10 min window in A. (C) A firing rate histogram of the neural activity in B was first constructed. (D) Then, the histogram was smoothed in latency with a Gaussian kernel, and all peaks (red circles) and valleys (green pentagons) were found (only 2 peaks and their corresponding valleys are plotted for clarity). A peak was considered to contain dAPs if it exceeded an empirical threshold.

For each recording electrode, detected electrically evoked spikes were sorted from peaks in a firing rate histogram (Fig. 7), and latencies were tracked in consecutive moving time windows throughout the duration of an experiment. The histograms were constructed from 10 min windows (140 probe stimuli), stepped by 1 min. A given histogram had a bin width of 0.04 ms, corresponding to the sampling frequency of 25 kHz, and was smoothed in latency with a Gaussian kernel size of 31 samples. All histogram peaks and valleys were found, up to 25 ms in latency. Directly-evoked action potentials (dAP) produced tall sharp peaks while synaptically-evoked action potentials (sAP) produced broader shallower peaks. Thus, a peak was considered to be caused by dAPs if the peak height was greater than 2 times the highest valley plus 0.5, which was an empirically determined threshold. The analysis was done separately for positive and negative height spikes. All assigned dAPs were verified manually in raster plots and by waveform.

To track changes in the latency of a dAP, the peaks in consecutive histograms were compared.

1. If an assigned peak overlapped a peak in the previous histogram within a tolerance, then the peaks were considered to arise from the same dAP. The tolerance was the width of the Gaussian at the peak's half height plus 440 µs (11 samples) on either side. The tolerance allowed tracking a dAP that changed latency.
2. If a peak did not overlap any prior peaks, then a new dAP was assigned.
3. On rare occasions, if a peak overlapped 2 prior peaks, then the new peak was matched to the closest prior peak.

Occasionally, dAPs would disappear and reappear. Therefore, a peak was kept in memory until overlapped by a new peak. However, only stable dAPs were considered in the paper.
